# Methicillin-resistant *Staphylococcus aureus* enterocolitis in a 16-month-old boy: a case report

**DOI:** 10.1186/s13256-022-03381-z

**Published:** 2022-04-17

**Authors:** Christopher Loerke, Heather Liebe, Catherine J. Hunter

**Affiliations:** 1 The University of Oklahoma College of Medicine, 800 Stanton L Young Blvd, Oklahoma City, OK 73117 USA; 2 Division of Pediatric Surgery, Oklahoma Children’s Hospital, 1200 Everett Drive, ET NP 2320, Oklahoma City, OK 73104 USA

**Keywords:** Methicillin-resistant *Staphylococcus aureus*, Enterocolitis, Vancomycin, Case report, Pediatric

## Abstract

**Background:**

Methicillin-resistant *Staphylococcus aureus* enterocolitis is a rare disease that typically affects immunocompromised adults. Most cases of pediatric enterocolitis are caused by Gram-negative bacteria, Gram-positive *Clostridiodes difficile*, or viruses. This is the first published case report of a toddler with methicillin-resistant *Staphylococcus aureus* enterocolitis.

**Case presentation:**

A 16-month-old non-Hispanic White boy with no past medical or psychosocial history initially presented to the emergency room with abdominal pain and emesis. Past family history was pertinent only for his father having a history of constipation. He was diagnosed with intussusception and underwent successful contrast reduction on hospital day 0. The following day, the patient had recurrent symptoms and a repeat contrast enema showed no evidence of recurrent intussusception. A computed tomography scan was obtained, which was concerning for possible recurrence with compromised bowel. He was taken to the operating room for operative reduction and underwent an ileocecetomy with primary handsewn end-to-end anastomosis. His postoperative course was complicated by an anastomotic leak on hospital day 6 necessitating reoperation and creation of an end ileostomy with mucous fistula. He received intravenous metronidazole, ceftriaxone, and ceftazidime antibiotics during his hospital course. On postoperative day 12, the patient developed a sudden increase in ileostomy output, and stool cultures were obtained. His symptoms persisted despite diet modifications, stopping antibiotics, and initiating loperamide. Three days later, stool cultures resulted negative for *Escherichia coli*, *Salmonella*, *Shigella*, *Campylobacter* species, and *Clostridiodes difficile* but were positive for methicillin-resistant *Staphylococcus aureus*. The patient was started on a 10-day course of oral vancomycin and discharged home in good condition 4 days later. After 12 weeks, the patient underwent reversal of the ostomy and is doing well at the 1 month postoperative follow-up, now 5 months from his initial surgery.

**Conclusions:**

To our knowledge, this is the first published report of a toddler being diagnosed with methicillin-resistant *Staphylococcus aureus* enterocolitis. Because methicillin-resistant *Staphylococcus aureus* enterocolitis is rare and has overlapping symptoms with more common gastrointestinal pathologies, it is often misdiagnosed. When a patient presents with diarrhea or high ostomy output along with fecal cultures negative for *Clostridiodes difficile* and other common pathogenic agents, methicillin-resistant *Staphylococcus aureus* should be considered.

## Background

Methicillin-resistant *Staphylococcus aureus* (MRSA) enterocolitis is a rare disease that typically affects immunocompromised adults [1–4]. While MRSA is a common cause of nosocomial infection, its colonization in the upper respiratory tract does not typically migrate to the gastrointestinal tract [5, 6]. Because signs and symptoms of MRSA enterocolitis include a pseudomembrane seen on colonoscopy, diarrhea, and abdominal pain, this illness is often misdiagnosed as *Clostridiodes difficile* infection [1]. The causes of enterocolitis in pediatric patients are typically Gram-negative bacteria, Gram-positive *Clostridiodes difficile*, or viruses. While case reports of MRSA enterocolitis in adults and infants have been published, this is the first case to be published of a toddler with MRSA enterocolitis [7–9]. We propose that an unexplained increase in ostomy output or frequent diarrhea should lead to stool cultures screening for MRSA alongside other common gastrointestinal pathogens.

## Case presentation

An otherwise healthy 16-month-old non-Hispanic White male with no past medical or psychiatric history presented to the emergency room with 2 days of abdominal pain and emesis. Family history was only positive for constipation in the patient’s father. The patient lived at home with his parents and sister and had no financial, language, or cultural challenges. Upon admission, the patient’s physical exam was within normal limits and, particularly, his abdomen was soft, nondistended, nontender to palpation with no masses. He was diagnosed with intussusception, which was successfully reduced by radiology via water-soluble contrast enema. The following day, he had persistent symptoms and was taken for repeat contrast enema to evaluate for recurrent intussusception. The contrast enema was negative for recurrence, and a computed tomography (CT) scan was obtained for further evaluation. CT scan did not demonstrate intussusception, but raised concern for small bowel obstruction with compromised bowel. At this time, the patient’s physical exam continued to be within normal limits, but he subjectively appeared uncomfortable with increased fussiness and agitation. Given the patient’s history, there was concern for recurrent intussusception requiring operative reduction. The patient underwent operative exploration and was found to have an intussusception in his terminal ileum. This was manually reduced but noted to have necrotic small bowel which was resected with a primary, end-to-end anastomosis, and an appendectomy was performed. The pathology report postoperatively described the specimen as being from the small intestine with ischemic necrosis along with an intraluminal fibrin and fragments of necrotic bowel mucosa.

The patient’s course was complicated by an anastomotic leak along with three small bowel perforations on hospital day 6 requiring an exploratory laparotomy with abdominal washout, small bowel resections, and temporary abdominal closure. Pathology showed segments of small intestine with patchy ischemic necrosis, serositis, and numerous transmural defects. He was taken back 2 days later for end ileostomy with mucous fistula creation. Total small bowel resected was 20–30 cm.

During his postoperative course, the patient was initially treated with IV ceftriaxone and metronidazole followed by ceftazidime and metronidazole. He was placed on total parenteral nutrition (TPN) on hospital day 9 and had return of bowel function on hospital day 10. By hospital day 19, antibiotics and TPN were stopped and he was advanced to a regular diet. On hospital day 20, he developed two episodes of nonbilious emesis and high ileostomy output at 50–60 mL per kilogram (kg) per day. At this time, the patient continued to have a normal abdominal exam. His ostomy was pink, patent, and productive. The patient received replacement fluids, and stool cultures were collected. Ileostomy output remained high despite interventions which included loperamide, modifying feeds, and correcting electrolyte abnormalities. During this time, the patient was otherwise asymptomatic, tolerating a diet, and had normal labs and physical exam. Stool cultures resulted on hospital day 24 and were negative except for a large growth of MRSA. The patient was diagnosed with MRSA enterocolitis and started on 13 mg/kg oral vancomycin three times daily. The patient tolerated this well, and his ostomy output normalized by hospital day 27. The patient was discharged home in good condition on hospital day 29.

A summary of the patient’s postoperative clinical course is as follows. He was seen by a gastrointestinal rehabilitation specialist 3 weeks after discharge, and no complications were noted. He was later seen in clinic by his pediatric surgeon 5 weeks after discharge with no complications and was deemed ready for ileostomy reversal. He underwent ileostomy reversal 12 weeks after discharge and is currently doing well now at his postoperative visit 5 months after his initial surgery.

Timeline of case progression:

**Figure:**
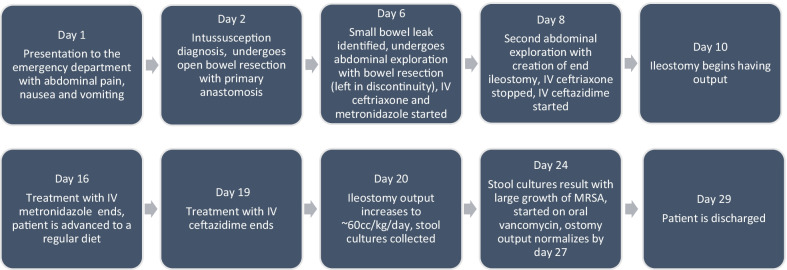

## Discussion and conclusions

Methicillin-resistant *Staphylococcus aureus* (MRSA) enterocolitis is a rare and devastating disease. While there are many documented cases of adults being diagnosed with MRSA enterocolitis, there are few pediatric cases. Specifically, there are no existing published reports of a toddler diagnosed with MRSA enterocolitis [7–9].

While MRSA is a common nosocomial infection, it is typically manifested in the skin with colonization in the upper respiratory tract [5]. Most cases of bacterial enterocolitis are caused by Gram-negative bacteria due to its abundance in the intestinal microbiome. MRSA enterocolitis is of particular interest because it is caused by an infection with an aerobic, Gram-positive organism and is found most often in immunocompromised adults. Common risk factors include old age, previous surgery, antibiotic use, and preexisting disorders affecting the immune system [1–4]. Because of the potential development of a pseudomembrane and the common symptom of diarrhea, MRSA enterocolitis is often misdiagnosed as *Clostridium difficile* infection [1]. Other reported symptoms include fever, abdominal pain, and malaise [3, 9]. In a retrospective study performed by Gravet *et al.* of 60 patients with MRSA associated diarrhea, 98% had been treated with one or more antibiotics within a month before symptoms appeared [4]. Gastrointestinal flora is a balance of primarily Gram-negative anaerobic bacteria that can be disrupted by antibiotics. This disruption results in an imbalance of bacteria, allowing the colonized MRSA to grow and overcome other, nonpathogenic bacteria, resulting in infection [9]. Elderly individuals and those who have undergone recent surgery not only have suppressed immune systems but also are often in environments (such as nursing homes and hospitals) where the risk of a MRSA outbreak is elevated [1–3].

While there is a lack of consensus on how MRSA colonizes the gut, evidence in the literature suggests that MRSA, already colonized in the upper respiratory tract, travels through the digestive system and, aided by antibiotics, grows opportunistically in the patient’s bowel [6]. The most frequently cited treatment for MRSA enterocolitis is oral vancomycin, but it is critical to reestablish a healthy gut microbiome containing primarily Gram-negative bacteria [9, 10]. This can be done by introducing probiotics and prebiotics such as *Bifidobacterium breve*, *Lactobacillus casei*, and galactooligosaccharides to the patient after treatment with antibiotics [9]. Studies have also supported reestablishing a balanced gut microbiome through a fecal microbiota transplant [10].

This case report underscores the importance of a broad differential and clearly demonstrates that increased ostomy output has the potential to be associated with MRSA enterocolitis. The limitations of this report include the patient’s complicated surgical and medical history, which may confound our conclusions.

In conclusion, MRSA enterocolitis is an uncommon form of enterocolitis and even more rare in the pediatric population. To our knowledge, there has not been another report published of a toddler with MRSA enterocolitis [7–9]. This disease is often misdiagnosed and carries a high mortality rate. An unexplained increase in ostomy output or frequent diarrhea should lead to stool cultures screening for MRSA alongside other common gastrointestinal pathogens. Early recognition and appropriate treatment are critical to decreasing the morbidity and mortality of this disease process.

## Data Availability

Data sharing is not applicable to this article as no datasets were generated or analyzed during the current study.

## Abbreviations

CT: Computed tomography
GI: Gastrointestinal
IV: Intravenous
kg: Kilogram
MRSA: Methicillin-resistant *Staphylococcus aureus*
TPN: Total parenteral nutrition
